# Computational Investigation of 4‑Nitrophenol Inclusion Complexes with α‑, β‑, and γ‑Cyclodextrins

**DOI:** 10.1021/acsomega.5c08868

**Published:** 2026-01-27

**Authors:** Laura Fernanda Osmari Vendrame, Mariana Zancan Tonel, João Augusto Pereira da Rocha, Ivana Zanella, Cristiano Rodrigo Bohn Rhoden, Solange Binotto Fagan

**Affiliations:** 1 Universidade Franciscana, Rua dos Andradas, 1614, Santa Maria, RS 97010-100, Brazil; 2 Instituto Federal de Educação Ciência e Tecnologia do Pará, Research Group BioInovaMol Amazônia, Campus Bragança, Av. dos Bragançanos-Vila Sinhá Bragança, Bragança, PA 68600-000, Brazil

## Abstract

The increasing presence of emerging contaminants, such as 4-nitrophenol (4-NP), in aquatic environments poses environmental and public health risks, driving interest in innovative systems capable of selectively removing them. Despite the well-established potential of cyclodextrins (CDs) as molecular hosts for the removal of organic micropollutants, owing to their ability to reduce contaminant mobility and availability while promoting capture, isolation, and preconcentration, as well as their biodegradability and low toxicity, and increasing their chemical versatility, a comprehensive theoretical comparison of their interactions with 4-NP is still lacking. The present study explores the formation and stability of inclusion complexes between 4-NP and three types of cyclodextrins (α-CD, β-CD, and γ-CD) using a combination of docking, molecular dynamics, and *ab initio* density functional theory (DFT) calculations. The results show that α-CD exhibits the strongest and most stable interaction with 4-NP, followed by β-CD. At the same time, γ-CD exhibits lower retention, consistent with a cavity–guest size mismatch and structural complementarity on molecular interactions. These findings not only provide molecular-level insights into host–guest interactions but also reinforce the potential application of cyclodextrins as effective, biodegradable, and reusable materials, offering theoretical support for their use in environmental remediation strategies and their potential for reuse, making them a sustainable and cost-effective solution.

## Graphical abstract

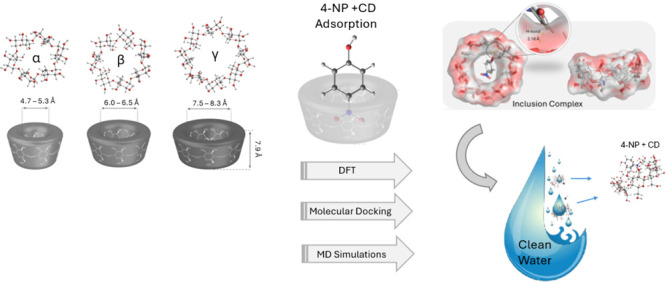

## Introduction

The growing scarcity of potable water, exacerbated by the presence of both organic and inorganic pollutants, represents one of the most urgent environmental challenges today.^1^ In this context, 4-NP, an aromatic compound widely used as an intermediate in the production of pesticides, dyes, and pharmaceuticals, has emerged as a persistent and highly toxic pollutant.^1,2^ As a priority pollutant that is highly toxic, chemically stable, moderately water-soluble, and nonbiodegradable, 4-NP exhibits characteristics that favor its dispersion, persistence, and difficulty of removal in aquatic environments.^9^ Its concentrations in wastewater and surface waters have been reported to range from 0.2 to 18 μg L^–1^ in several monitoring studies, often exceeding recommended limits due to its persistence and low biodegradability.^1^ Similar occurrence ranges have been summarized in recent reviews addressing contaminants of emerging concern.^3^ These properties make 4-NP a model contaminant for assessing molecular recognition and removal mechanisms using sustainable adsorbents.^4^

Consequently, the removal of Contaminants of Emerging Concern (CECs) has attracted the attention of researchers seeking effective and sustainable solutions.^3^ In this context, α-CD, β-CD, and γ-CD have emerged as promising alternatives due to their ability to form inclusion complexes through host–guest inclusion of pollutants, such as 4-NP. By forming inclusion complexes, CDs can reduce the mobility, availability, and interaction of 4-NP with the surrounding medium, promoting its capture, isolation, and preconcentration. This ability to modify the pollutant’s behavior in solution supports the growing interest in understanding their supramolecular interactions and potential in environmental remediation and monitoring applications. The three naturally occurring cyclodextrins α-, β-, and γ-CD consist of six, seven, and eight glucose units, respectively, forming truncated-cone cavities with hydrophobic interiors and hydrophilic exteriors. Their internal diameters (≈4.7, 6.0, and 7.5 Å) increase with ring size, allowing selective inclusion of guest molecules of different dimensions.

Although 4-nitrophenol (4-NP) shows moderate water solubility due to its polar hydroxyl and nitro groups, the aromatic ring retains hydrophobic character that enables partial inclusion within the nonpolar cyclodextrin cavity, while the polar substituents remain near the rim forming hydrogen bonds with hydroxyl groups.

Recent studies highlight the relevance of investigating the interaction between 4-NP and cyclodextrins, emphasizing that the supramolecular selectivity of these host molecules is essential for analytical and environmental applications aimed at monitoring and mitigating phenolic pollutants. This reiterates the difficulty of removing 4-NP from aquatic environments due to its dispersive, persistent, and nonbiodegradable nature. In addition to emphasizing that CDs provide selective recognition and the ability to form inclusion complexes with aromatic compounds such as 4-NP.^9^

In addition to their high selectivity and operational simplicity, cyclodextrins offer significant potential for reuse, making them promising materials for advanced water purification technologies.^5,6^

Recent studies have explored cyclodextrin-based strategies as versatile adsorbents for water and wastewater treatment, with reports highlighting their ability to recognize and capture persistent organic pollutants (POPs), metal ions, phenolic pollutants (e.g., 4-nitrophenol), pharmaceuticals, and other organic contaminants.^7–10^

From a theoretical standpoint, combining molecular docking with molecular dynamics has proven effective in elucidating how α-, β-, and γ-CD encapsulate environmentally relevant contaminants, clarifying preferred binding modes, complex stability, and the contributions of hydrophobic, dispersion, and hydrogen-bonding interactions.^11,12^ *Ab initio* DFT calculations have likewise examined β-cyclodextrin inclusion complexes with substituted phenolic guests, showing that the cyclic cavity of β-CD promotes encapsulation and stabilizes the host–guest complex, potentially lowering the guest’s apparent bioavailability.^13,14^

Recent computational works have applied noncovalent interaction (NCI) and free energy perturbation (FEP) approaches to investigate host–guest stabilization in cyclodextrin inclusion complexes, providing refined insights into the energetic and electronic properties.^15–17^

Considering these factors, this study investigates the interactions between 4-NP and α-, β-, and γ-CD, employing theoretical methodologies such as molecular docking, molecular dynamics, and *ab initio* calculations to provide a detailed understanding of the complexes formed and their stability in aqueous systems. To date, few studies have compared the interactions of all three cyclodextrin forms, α, β, and γ, with contaminants using a combined set of theoretical techniques. This integrative approach enables a comprehensive evaluation of host–guest inclusion, stability, and electronic structure across different cavity sizes, thus filling a significant gap in the current understanding of 4-NP encapsulation by cyclodextrins. Our analysis, therefore, offers an original contribution to elucidating the complexation mechanisms involved. These studies are crucial for understanding how cyclodextrins can be used in the removal of pollutants from water and soil.

## Materials and Methods

### Ab Initio Calculations

We evaluated the interaction of 4-NP with cyclodextrins through first-principles calculations based on DFT^18,19^ to obtain the electronic, structural, and energetic properties. We used the SIESTA program,^20^ which performs self-consistent calculations by solving the spin-polarized Kohn–Sham equations using sets of atomic numerical orbital bases. The double-ζ base plus a polarized function (DZP) is applied to describe the pseudo-orbitals in all simulations. 200 Ry of the cutting grid was used to represent the electronic charge in real space. A local density approximation (LDA) was employed for the exchange and correlation potential, utilizing the parametrization of Perdew and Zunger.^21^ All atomic structures were relaxed until all atoms had less than 0.01 eV/Å residual forces. The self-consistent field (SCF) cycles were converged until the total energy difference between successive iterations was smaller than 10^–4^ eV.

To calculate the binding energy, we used the Basis Set Superposition Error correction (BSSE),^22^ calculated according to the equation below:

$$E_{b}=\left(E_{A+B}-E_{A_{ghost}+B}-E_{A+B_{ghost}}\right)$$

System *A*{*B*} corresponds to 4-NP­{CD}. The energy values are all calculated based on their respective atomic bases, with the atomic base *A*{*B*} centered on the atomic positions *B*{*A*}. The ghost refers to the atomic base placed at the 4-NP or cyclodextrins positions, but without atomic potentials representing real atoms at those positions. The group has already published a similar methodology system.^23,24^

### Molecular Docking

Molecular docking was performed using AutoDock Vina^25,26^ to explore the interactions between 4-NP and α-CD, β-CD, and γ-CD. The molecular structures of all compounds were obtained from the PubChem database (4-NP: CID 980; α-CD: CID 444913; β-CD: CID 444041; γ-CD: CID 5287407) and, subsequently, geometry-optimized with Gaussian to obtain low-energy conformations before docking.

The docking calculations used a cubic grid box (40 × 40 × 40 Å^3^) centered on the geometric centroid of each cyclodextrin, encompassing the internal cavity and allowing exploration of both interior and exterior binding sites. This box size accommodates the different cavity diameters of α-/β-/γ-CD and permits unrestricted sampling of 4-NP orientations relative to the host, similar to studies already carried out in our research group.^27^ Cyclodextrin was treated as rigid, while the 4-nitrophenol ligand was prepared with one rotatable bond corresponding to the C–N connection of the nitro group to the aromatic ring. This limited torsional degree of freedom is consistent with the conjugated nature of the molecule, since all other single bonds are either part of the aromatic system or terminal (O–H).

For each system, different poses were generated, and the results were ranked by the Vina score (kcal/mol^–1^). The arrangements were then inspected in PyMOL^28^ and Discovery Studio Visualizer.^29^ The best-scoring conformation within the cavity was selected considering both the predicted affinity and a chemically reasonable orientation, characterized by the insertion of the aromatic ring of 4-NP into the CD cavity and the formation of hydrogen bonds between the 4-NP and the rim hydroxyls of the cyclodextrin. In addition to binding affinity, the selected poses were further analyzed for noncovalent interactions, including hydrogen bonding, hydrophobic interactions, and π–π stacking, to achieve deeper insights into the stabilization mechanisms of the inclusion complexes.

### Molecular Dynamics

The complexes for molecular dynamics studies are formed by α-CD, β-CD, and γ-CD with 4-NP using the previously obtained configurations from molecular docking. The systems were parametrized with the GLYCAM06 force field, specialized in modeling glycosidic interactions.^30^ For 4-NP, the Generalized Amber Force Field (GAFF) force field was employed to ensure an accurate description of the interactions between the ligand and the solvent, with the parameters being optimized using Antechamber.^31,32^

The partial charges of 4-NP were assigned using the Restrained Electrostatic Potential (RESP) method^33^ in Gaussian at the HF/6-31G­(d) level.^34^ The complexes were solvated in a cubic box of TIP3P water.

Prior to heating, each system underwent energy minimization in four stages, progressively releasing positional restraints. The heating simulation was performed from 0 to 300 K for 100 ps, followed by a 500 ps equilibration period at 300 K. The production simulation was conducted for 100 ns using the AMBER 2023 simulation package, with temperature control at 300 K and pressure at 1 atm. Trajectories were recorded every 2 ps.^35,36^

The dynamics of hydrogen bonds were monitored using the “hbond” command of the CPPTRAJ module, and the distances between the center of mass of 4-NP and the cyclodextrins were calculated over time. Hydrogen-bond counts were monitored throughout the 100 ns trajectories, considering a donor–acceptor distance ≤3.5 Å and angle ≥135°. Average values and standard deviations were computed from the equilibrated portion of each trajectory. The three-dimensional trajectories were analyzed to identify movement patterns, preferred interaction regions, and specific dynamic behaviors, such as the entry and persistence of the ligand in the cyclodextrin cavity.

Binding free energy estimations were performed using the Molecular Mechanics Generalized Born Surface Area (MM-GBSA) method, as implemented in the AMBER 2023 suite. Trajectory snapshots were extracted from the last 20 ns of the 100 ns molecular dynamics simulations at 100 ps intervals.^37^

The production phase employed particle mesh Ewald (PME) for long-range electrostatics with a 10 Å real-space cutoff, SHAKE constraints on bonds involving hydrogens (allowing a 2 fs time step), and a Langevin thermostat (γ = 1 ps^–1^) combined with a Berendsen barostat. Coordinates were saved every 2 ps, and MM-GBSA binding free energies were calculated over the last 20 ns of each trajectory (200 frames per system). The entropic term (−*T*Δ*S*) was not included because, for this homologous α/β/γ-CD series with a common guest, enthalpic contributions provide a reliable and reproducible ranking; normal-mode or quasi-harmonic entropy estimates typically introduce high variance with limited effect on relative affinities.

## Results and Discussion

### Ab Initio Calculations

We evaluated the electronic and structural properties of isolated 4-NP and α-CD, β-CD, and γ-CD (Figure 1). The calculated HOMO (highest occupied molecular orbital)–LUMO (lowest unoccupied molecular orbital) energy difference (Δ*H*/*L*) for α-CD, β-CD, and γ-CD were 4.06, 5.73, and 4.96 eV, respectively, in agreement with values previously reported in the literature.^21–38^ Structural characterization further revealed cavity diameters of ∼4.7–5.3 Å/ 6.0–6.5 Å/ 7.5–8.3 Å for α-CD, β-CD, and γ-CD, respectively, consistent with experimental and theoretical studies.^39,40^ These variations in cavity size are directly related to the distinct inclusion capacities of each cyclodextrin, as widely discussed in prior investigations.^41^

**Figure 1:**
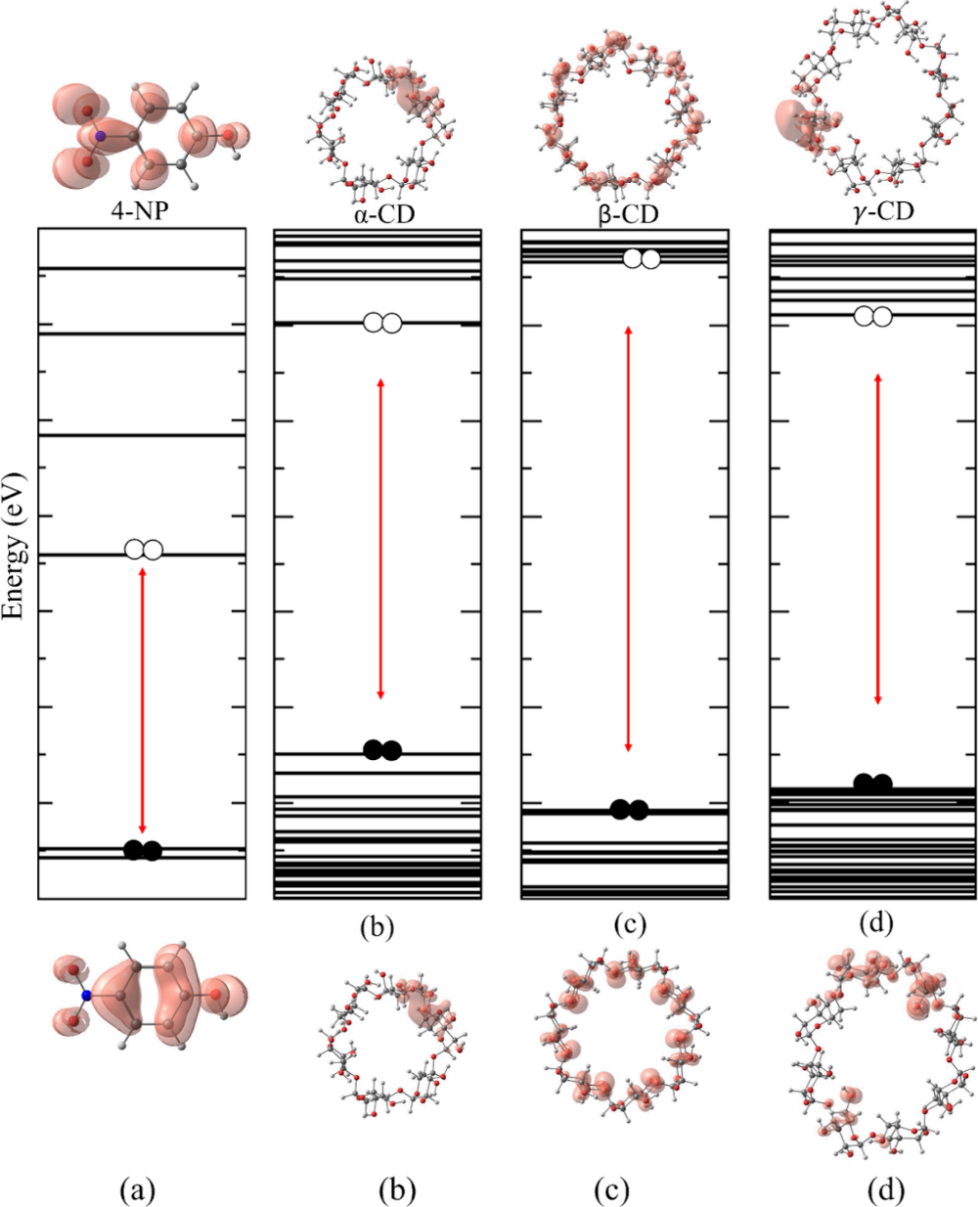
Isolated structure: 4-NP (a), α-CD (b), β-CD (c), and γ-CD (d). Well-defined energy levels with the charge distribution shown in the highest occupied molecular orbital (HOMO, at the bottom of the energy level) and the lowest unoccupied molecular orbital (LUMO, at the top of the energy level), respectively. Isosurface value used: 0.001 e^–^/Å^3^.

The electronic charge on α-CD is located on both HOMO and LUMO in a lateral region of the cyclodextrin. In the case of β-CD, the charge in the HOMO orbital is concentrated on the oxygen and carbon atoms, while the LUMO is located on the oxygen and hydrogen atoms. Finally, in γ-CD, the charge in HOMO is situated in the two most curved regions and in LUMO in only one of them, as already described in the literature.^21–33^ Cyclodextrins consist of glucopyranose units forming a relatively hydrophobic inner cavity lined mainly by C–H groups and glycosidic oxygen atoms, and a hydrophilic exterior due to primary and secondary hydroxyls.^42^

The most notable feature of cyclodextrins is their ability to form solid inclusion complexes (host–guest complexes) with a wide range of compounds by molecular complexation.

The 4-NP compound exhibits a Δ*H*/*L* value of 3.07 eV, consistent with findings previously reported in the literature employing comparable methodologies.^43,44^ The charge distribution in the HOMO is predominantly localized on the electron-rich carbon atoms of the aromatic ring, in association with the electronegative oxygen atoms of the single-bonded −NO_2_ and −OH groups, which contributes to a highly negative energy value.

Conversely, the LUMO is mainly delocalized over the electron-deficient nitrogen atom of the single-bonded −NO_2_ group, as well as the carbon atom bound to the electronegative −OH substituent, particularly on specific carbon sites. The negative LUMO energy indicates that the molecule exhibits a strong electron-accepting character, a behavior that has also been documented in prior studies.^43^

For the interaction of 4-NP with α-CD, β-CD, and γ-CD, several configurational arrangements were initially explored by positioning the guest molecule within both the primary and secondary cavities of each cyclodextrin host. To ensure reliability, all complexes were fully optimized, and their relative stabilities were compared. In the present study, we report only the most stable host–guest configuration for each system (Figure 2). The computed results are summarized in Table 1, which includes the binding energy (eV), electronic charge transfer (e^–^), shortest intermolecular distance (Å), and HOMO–LUMO energy difference (Δ*H*/*L*, eV), providing insights into both the stability and the electronic structure of the complexes.

**Table 1:** Interaction between α-, β-, and γ-CD with 4-NP: Binding Energy, Electronic Charge Transfer, Distance, and HOMO/LUMO Difference

	binding energy (eV/(kcal/mol))	charge transfer (e^–^)	distance (Å)	(Δ*H*/*L*)(eV)
α-CD@4-NP	–1.04/(−23.98)	–0.16	2.10	1.43
β-CD@4-NP	–0.99/(−22.83)	0.21	1.56	2.92
γ-CD@4-NP	–1.03/(−23.75)	0.06	1.63	2.12

**Figure 2:**
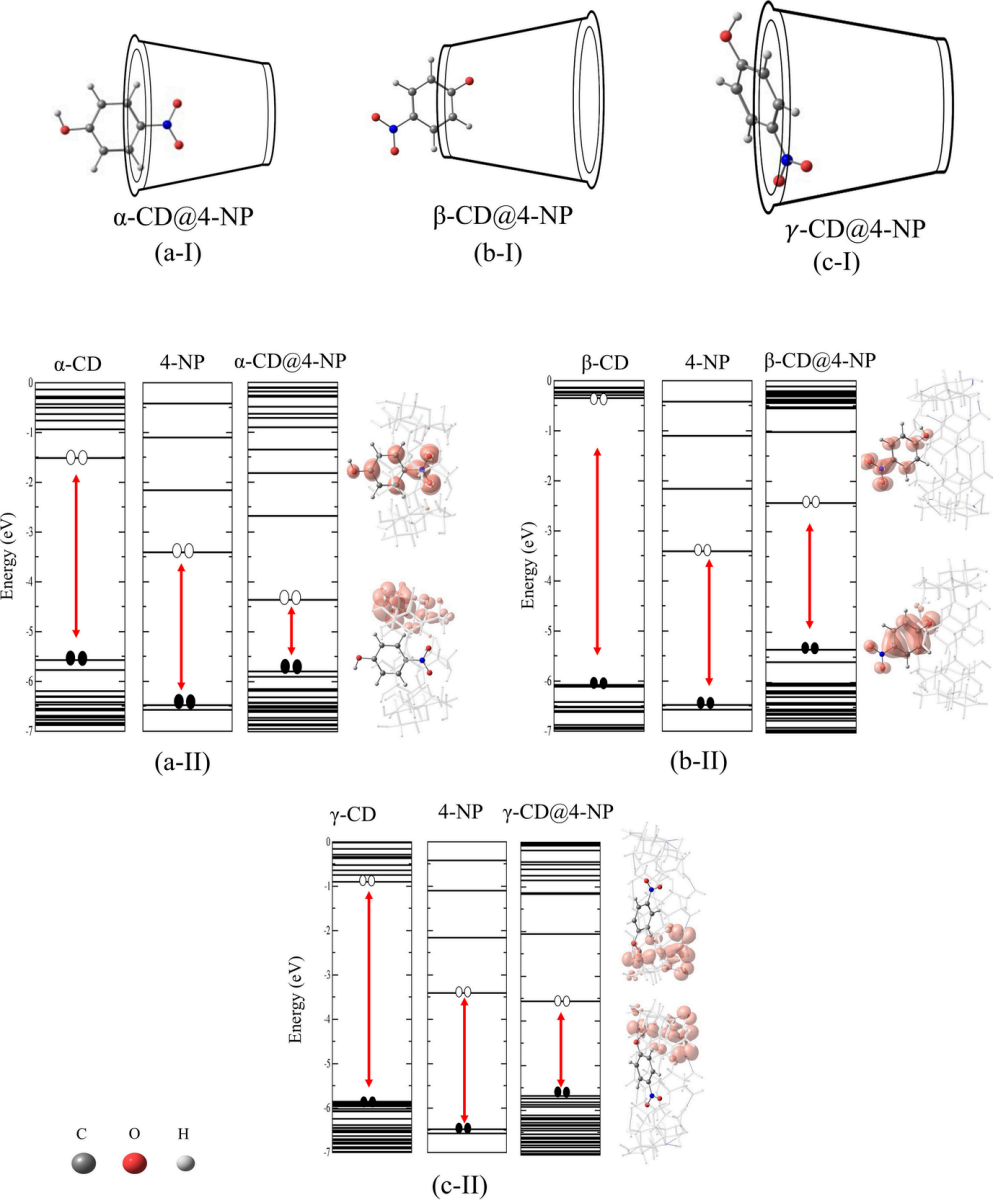
Most stable configurations of 4-NP interact with α-CD (a-I), β-CD (b-I), and γ-CD (c-I), along with the corresponding electronic charge distributions mapped onto the HOMO and LUMO. The applied isosurface value was 0.001 e^–^/Å^3^. The red line in the energy levels indicates the Δ*H*/*L*.

The 4-NP molecule has an average molecular size of 6.30 Å. In the present study, different configurational arrangements of cyclodextrins were evaluated, considering both internal and external orientations relative to the host cavity. The complexation of guest molecules with cyclodextrins is influenced by multiple factors, particularly the hydrophobic encapsulation of small nonpolar molecules within the internal cavity of the host. The main stabilizing interactions in these inclusion processes are hydrogen bonding, van der Waals forces, and hydrophobic effects.^45^

Additionally, the steric complementarity between the cavity size of the cyclodextrin and the dimensions of the guest molecule plays a crucial role in determining the stability of the complexes. Previous reports have shown that small, single-ring aromatic compounds strongly associate with α-CD,^33,46,47^ whereas larger aliphatic and hydrophobic aromatic compounds display a greater affinity for β-CD.^21,22^ In the case of γ-CD, its wider cavity allows the formation of ternary complexes, in which two small guest molecules can be simultaneously accommodated within its core.^48,49^

Different possible interaction modes of 4-NP with α-CD were evaluated, including inclusion within the cavity, orientations parallel to the cavity axis, perpendicular orientations at the entrances of the major and minor rims, and external binding. The most stable configuration was obtained when 4-NP is oriented perpendicularly and partially inserted into the larger cavity of α-CD. In this arrangement, the calculated binding energy was −1.04 eV (−23.98 kcal/mol), accompanied by a charge transfer of −0.16 e^–^ from 4-NP to α-CD. These results are consistent with the experimental observations reported by Cramer et al.,^45^ who demonstrated the inclusion of the aromatic ring of 4-NP within α-CD.

The electronic charge density distribution (Figure 2) further supports this stabilization mechanism. In the HOMO, the electronic density is predominantly localized on α-CD, whereas in the LUMO, it is mainly distributed over 4-NP. This electronic behavior highlights a preferential stabilization pathway in which α-CD acts as an electron donor while 4-NP assumes an electron-accepting role.

Such a trend is in agreement with previous reports indicating that aromatic guests, such as nitrophenols, generally display stronger affinity toward cyclodextrins when in their charged form compared to their neutral counterparts.^50^ Moreover, several studies corroborate the efficient accommodation of 4-NP within the α-CD cavity, reinforcing the structural and energetic viability of the complex.^51,52^

The most stable configuration of 4-NP with β-CD closely resembles that observed for α-CD. In this case, 4-NP adopts a perpendicular orientation and is partially accommodated within the β-CD cavity. The calculated binding energy for this configuration was −0.99 eV (−22.83 kcal/mol), accompanied by a charge transfer of 0.21 e^–^ from 4-NP to β-CD. The frontier orbital distribution reveals that the HOMO is primarily localized on β-CD, whereas the LUMO is concentrated on 4-NP. The resulting Δ*H*/*L* value of 2.92 eV indicates a reduction relative to the isolated species, consistent with the stabilization of the inclusion complex.

In the input by the electronic structures, we observed that the individual levels are maintained, with only an overlap. This fact shows that, despite the binding energy value, the interaction exhibits the characteristic of physical adsorption, i.e., a weak interaction. The β-CD cavity, acting as a host, can include 4-NP through hydrogen bonding interactions. No covalent bonds are broken or formed during the formation of an inclusion complex.^48,53^ Experimental studies^54^ show that 4-NP tends to be incorporated into β-CD. Furthermore, β-CD can be used in 4-NP adsorption/desorption cycles.^54^

The interaction of 4-NP with γ-CD exhibited features distinct from those observed with the other cyclodextrins. In the most stable configuration, 4-NP preferentially associates with the outer surface of γ-CD rather than being fully encapsulated within the cavity. The stabilization arises predominantly from dispersion forces and CH−π contacts with the glucopyranose framework, rather than from proper π–π stacking. In this configuration, the calculated binding energy was −1.03 eV (−23.75 kcal/mol), accompanied by a small charge transfer of 0.06 e^–^ from γ-CD to 4-NP. The HOMO distribution (Figure 2) remains primarily localized on 4-NP, whereas in γ-CD, the electronic density is concentrated in the interaction region, consistent with the noncovalent interactions governing the stability of this complex.^55^

The interaction of 4-NP with α-, β-, and γ-CD demonstrates that all host–guest complexes are primarily stabilized through weak noncovalent interactions. In the case of α-CD, 4-NP is partially accommodated within the cavity in a perpendicular orientation, exhibiting a binding energy of −1.04 eV (−23.98 kcal/mol) and a charge transfer of 0.16 e^–^. For β-CD, the enhanced stability arises from its favorable steric complementarity in conjunction with the synergistic contributions of hydrogen bonding, van der Waals forces, and hydrophobic interactions. Conversely, in γ-CD, the enlarged cavity promotes external binding, primarily through π–π stacking and C–H/O–H contacts, with the HOMO localized on 4-NP and the electron density of γ-CD concentrated in the interaction region. These observations align with well-established adsorption mechanisms of phenolic compounds, such as donor–acceptor interactions, dispersion forces, electrostatic attractions, and van der Waals contributions, which collectively rationalize the relative stabilities of the cyclodextrin inclusion complexes.^52^

### Molecular Docking

Molecular docking simulations of α-, β-, and γ-CDs were performed to evaluate the accommodation of 4-NP within the cavities and to analyze the interaction mechanisms, including binding modes and Vina affinity scores. The ligand was able to insert into all cyclodextrin cavities, although its orientation varied depending on the type of cyclodextrin. The most stable binding poses, defined by the lowest Vina affinity (docking score) and the most favorable spatial arrangement, are illustrated in Figure 3 for each complex: (a) 4-NP@α-CD, (b) 4-NP@β-CD, and (c) 4-NP@γ-CD. The predicted binding affinities (Vina scores) were −4.3, −3.8, and −3.5 kcal·mol^–1^ for α-, β-, and γ-CD, respectively. In addition, contacts compatible with hydrogen bonding were identified in the 4-NP@CD complexes, with interatomic distances of 2.18 Å for 4-NP@β-CD, 2.29 Å for 4-NP@α-CD, and 1.99 Å for 4-NP@γ-CD.

**Figure 3:**
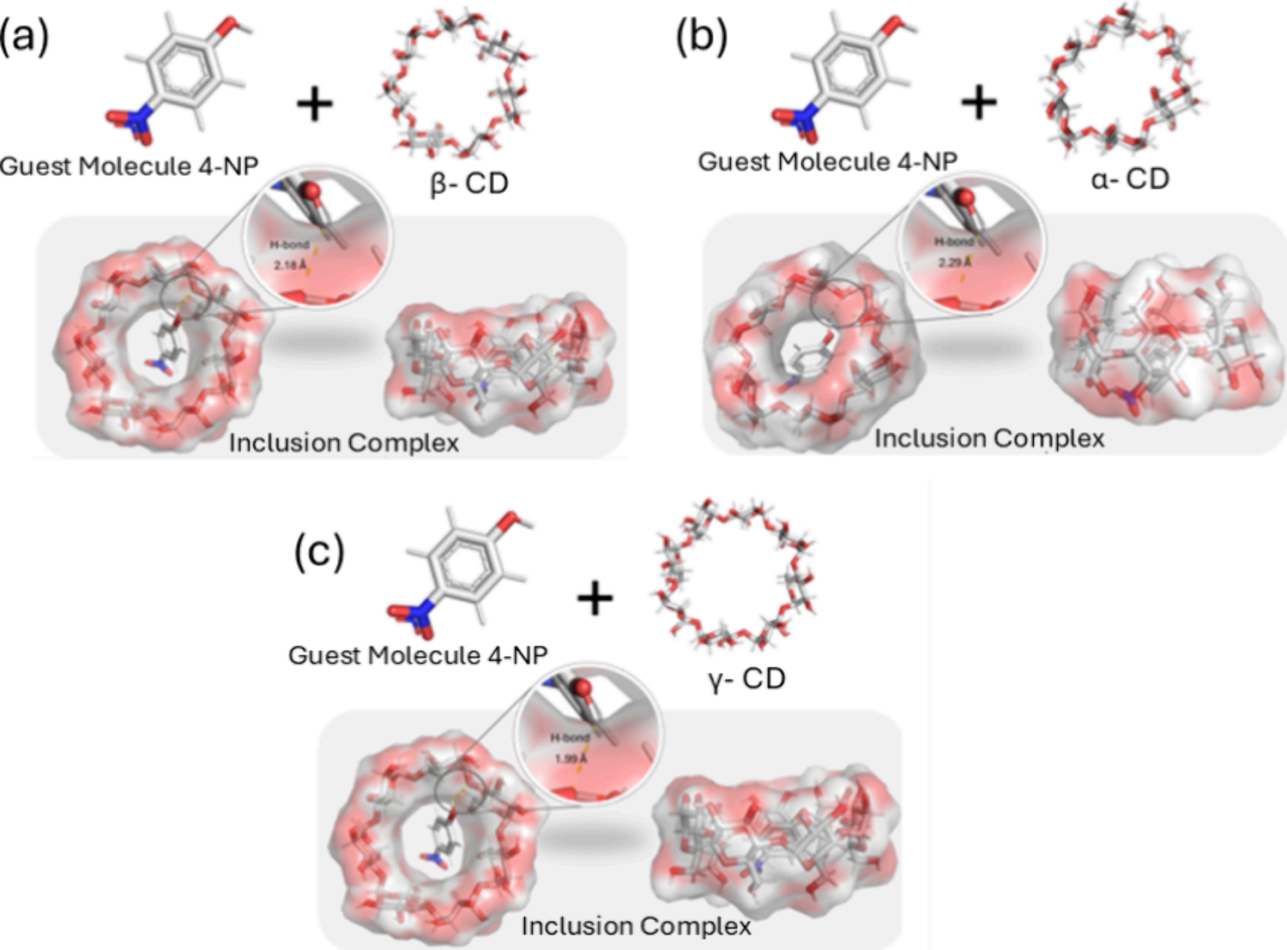
Best binding poses of 4-NP within the cavities of (a) β-CD, (b) α-CD, and (c) γ-CD, as determined by molecular docking simulations. The α-, β-, and γ-CDs can be observed from both lateral and top views, providing both lateral and top-down visualizations. Hydrogen bonds are indicated in the images, with calculated bond lengths of 2.29, 2.18, and 1.99 Å for α-CD, β-CD, and γ-CD, respectively.

These results indicate that all cyclodextrins could potentially accommodate 4-NP, with the interaction with α-CD being the most favorable, as indicated by the lowest (i.e., most negative) Vina affinity. This behavior can be attributed to differences in cavity size and structural compatibility between the cyclodextrins and 4-NP, which influence the ability of the guest molecule to fit and interact within each cyclodextrin’s cavity.

These findings are consistent with recent experimental and theoretical studies that support the ability of cyclodextrins to form inclusion complexes with phenolic compounds. Experimental studies have demonstrated that 4-NP can form inclusion complexes with β-cyclodextrin, as confirmed by FT-IR and Raman analyses.^9^ Although β-CD has been shown experimentally to interact effectively with 4-NP, our theoretical calculations indicate that α-CD provides the most favorable stabilization, suggesting that cavity size and host–guest fit play a key role in determining the inclusion strength across the CD family.

Additionally, theoretical studies involving structurally similar phenolic compounds, such as eugenol and chalcones, have demonstrated that these molecules are readily accommodated within the β-CD cavity and stabilized by noncovalent interactions, including van der Waals forces and hydrogen bonding.^57,58^ In addition to the calculated Vina affinity, the docking results were supported by the identification of key interactions, such as hydrogen bonds, which also contribute to the stabilization of the inclusion complexes. These findings were further explored through molecular dynamics simulations, as described in the following section.

### Molecular Dynamics

Molecular dynamics simulations provided insights into the dynamic behavior and intermolecular interactions of the studied systems. To assess complex stability, the center of mass distance between 4-NP and each cyclodextrin was monitored over 100 ns (Figure 4, left). The three-dimensional trajectory of 4-NP relative to each host is shown for context (Figure 4, right).

**Figure 4:**
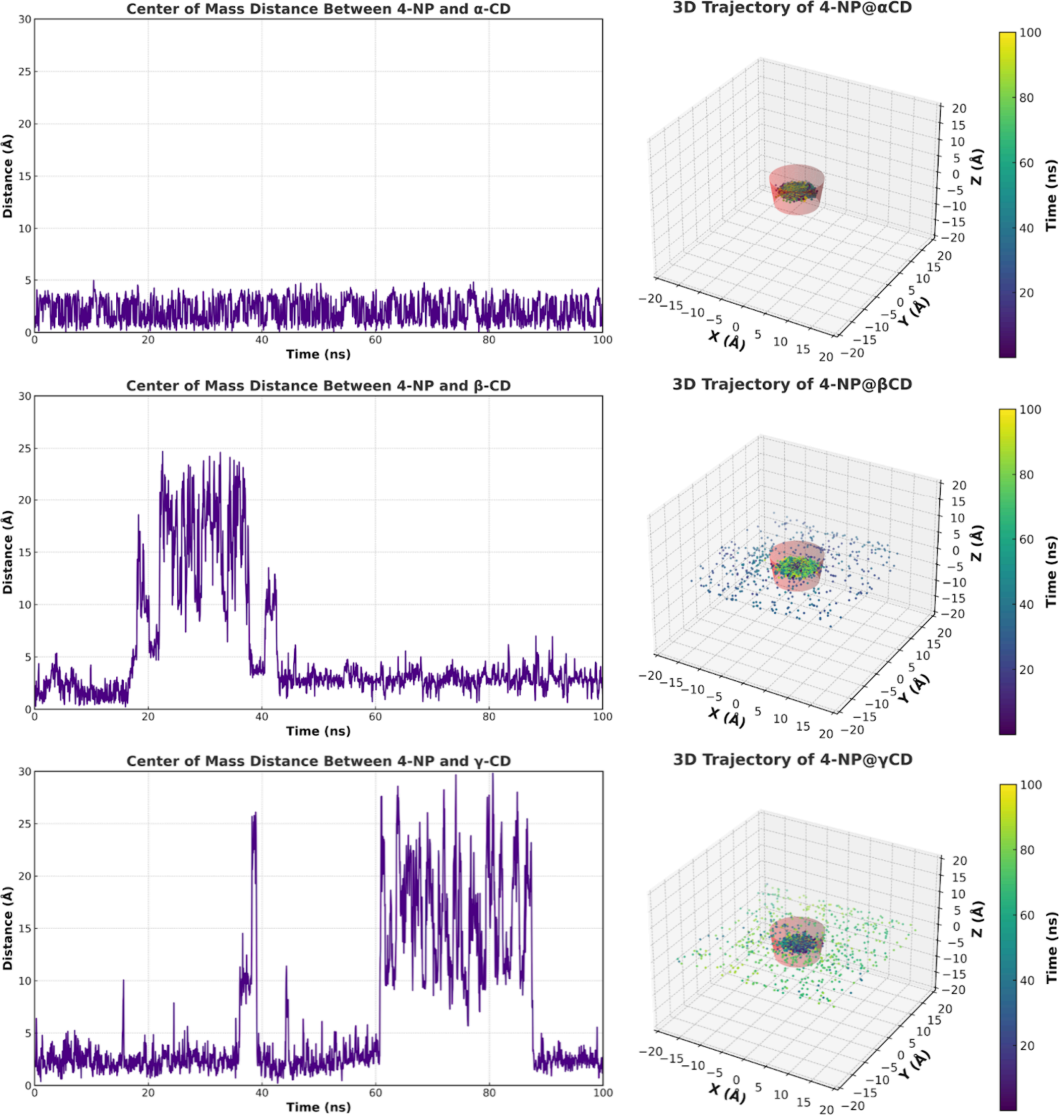
Center of mass of the central structure of the cyclodextrins was adopted as the origin of the graphs. The position of the 4-NP molecule along the *z*-axis is thus expressed in positive or negative values, indicating whether it is located above or below this reference point. To represent half the vertical height of the central cavity in the upward and downward directions, black dashed lines were included in the graphs. A displacement of the 4-NP molecule beyond these boundaries is indicative of its departure from the cyclodextrin cavity.

Figure 5 presents the *z*-axis position of 4-NP with respect to the cyclodextrin center. In this plot, the red dashed lines mark the cavity boundaries, and crossings indicate that the guest is outside the cavity volume.

**Figure 5:**
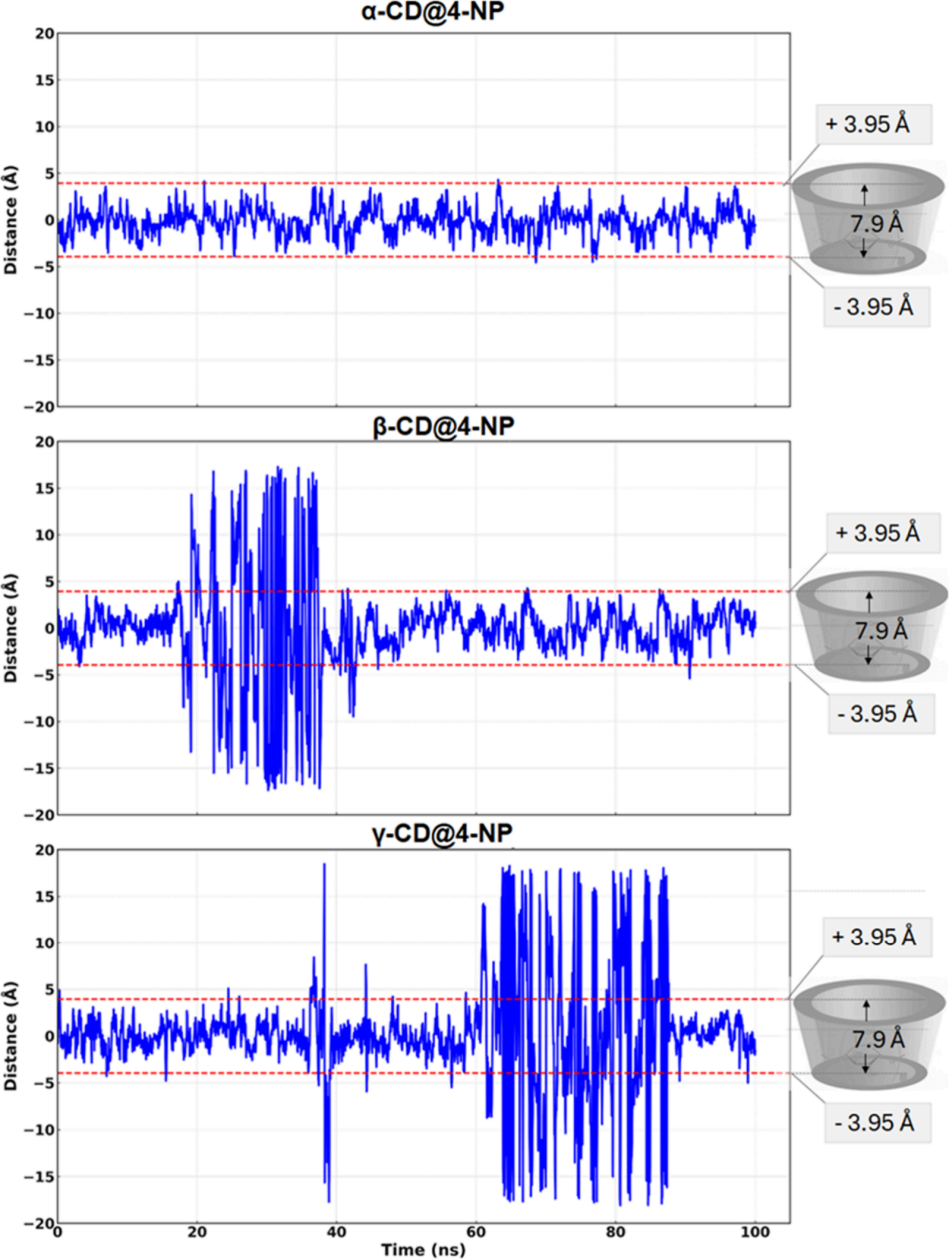
*Z*-axis distance between the center of mass of 4-NP and the core of α-, β-, and γ-CD during 100 ns of simulation. The origin corresponds to the center of mass of the cyclodextrins, with positive or negative values indicating the position of 4-NP above or below this point. The red dashed lines mark the boundaries of the cyclodextrin cavities; crossing them indicates molecular exit. The truncated-cone diagrams on the right represent the total vertical dimension of the central cyclodextrin structures.

For 4-NP@α-CD, the center of mass trace shows no sustained excursions or signs of dissociation, indicating that the ligand remains confined to the host region throughout the run. Consistently, in Figure 5, the 4-NP coordinate oscillates within the dashed-line limits, without persistent crossings into the solvent phase, a pattern compatible with internal reorientations rather than detachment.

For 4-NP@β-CD, after an initial bound interval, the center of mass trace exhibits an intermediate segment with larger values, followed by a return to lower distances. In Figure 5, this phase coincides with the crossings of the *z*-axis cavity limits, and the 3D trajectory shows a more dispersed point cloud near and above the rim. However, toward the end, the positions are again concentrated inside the cavity, consistent with the transient egress and subsequent reassociation of the guest. These observations are in agreement with prior reports of temporary egress/reentry in β-CD inclusion complexes.^56^

For 4-NP@γ-CD, the system is the most transient; the center of mass distance alternates between bound segments and prolonged excursions (Figure 4, left). The *z*-axis analysis reveals frequent and extended crossings beyond the dashed boundaries (Figure 5), and the 3D trajectory encompasses widespread positions outside the cavity, with intermittent returns to the interior, consistent with the reduced retention of a small aromatic guest in the larger γ-CD cavity.

Overall, Figures 4 and 5 support a clear trend: α-CD maintains a stable inclusion, β-CD shows temporary egress and reentry, and γ-CD displays intermittent association with longer solvent-exposed intervals.

Figure 6 shows the time evolution of the number of hydrogen bonds between 4-NP and each cyclodextrin. In α-CD@4-NP, a sustained hydrogen-bonding pattern is observed for most of the simulation, with only brief fluctuations, consistent with a stable inclusion state maintained. β-CD@4-NP shows fluctuations in the number of hydrogen bonds, reflecting variable host–guest interactions along the trajectory. γ-CD@4-NP displays the most transient behavior, including a prolonged interval with near-zero bonds, followed by reassociation toward the end of the run. This trend agrees with the center of mass and *z*-axis analyses (Figures 4 and 5).

**Figure 6:**
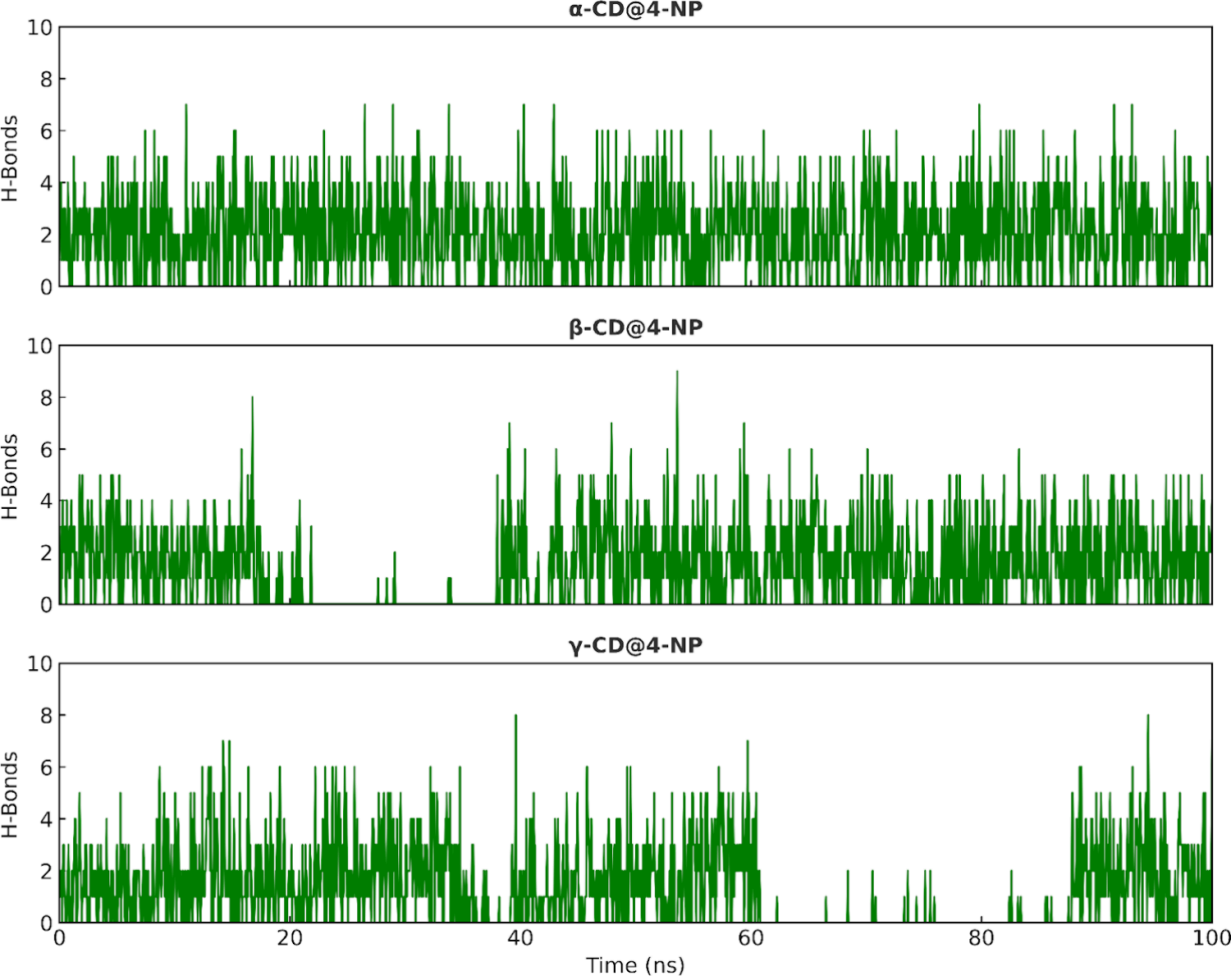
Time evolution of the number of hydrogen bonds (H-bonds) between 4-NP (guest) and the cyclodextrin (host) during the molecular dynamics simulation.

Finally, we also performed MM-GBSA free energy calculations to estimate the binding affinities of γ-, β-, and α-CD toward 4-NP. MM-GBSA is a widely used postprocessing method that combines molecular mechanics energies with solvation terms to provide an efficient approximation of binding free energies from molecular dynamics trajectories. Despite their limitations, such as the absence of entropic contributions and sensitivity to conformational sampling, these inputs offer a valuable balance between computational cost and accuracy for comparing similar host–guest systems. The results indicate that 4-NP binds most strongly to α-CD, with a binding free energy of −12.50 ± 2.4277 kcal mol^–1^. In comparison, the binding energy for β-CD is −8.64 ± 4.5389 kcal mol^–1^, and for γ-CD, it is −7.17 ± 4.6088 kcal mol^–1^.

These values provide a quantitative benchmark for evaluating and optimizing host–guest interactions in cyclodextrin-based systems, revealing that the behavior of 4-NP varies considerably as a function of the cavity size. Thus, α-CD enables closer contact with the ligand, promoting stronger interactions, while β-CD offers a balance between structural stability and effective encapsulation. In contrast, γ-CD, with its larger cavity, exhibits weaker retention and reduced stability, which may limit its suitability for specific applications.

Our results indicate that inclusion stability is favored by π–π stacking and hydrogen-bonding interactions between the aromatic ring of 4-NP and the cyclodextrin cavity. Thus, derivatives bearing additional hydrophobic or aromatic substituents could enhance guest encapsulation through stronger dispersion and π–π contacts. Furthermore, controlling the ionization state would favor host–guest association, providing practical insight for optimizing cyclodextrin-based remediation systems.

Recent experimental studies have examined the inclusion of 4-nitrophenol within α-, β-, and γ-cyclodextrins by FT-IR and Raman spectroscopy, providing evidence of 1:1 host–guest complex formation.^9^ The most pronounced spectral changes, particularly near 1585 and 1325 cm^–1^, were observed for β-CD, whereas α- and γ-CD exhibited weaker or more external interactions. FT-IR and Raman analyses suggested that the NO_2_-substituted aromatic ring interacts with all three hosts, with a deeper insertion into the β-CD cavity.^9^ Although our theoretical calculations predict the stability trend α > β > γ, both approaches consistently identify γ-CD as the least favorable host and indicate stronger stabilization for α- and β-CD. These findings underscore the qualitative agreement between theoretical predictions and experimental observations within their respective environments.

The use of LDA in DFT and the approximate nature of MM-GBSA methods and the limited simulation time may impose minor constraints. Future studies should include extended simulations and additional experimental measurements to further validate and refine the theoretical predictions presented here.

## Conclusions

In this study, we employed a combination of molecular docking, molecular dynamics, and *ab initio* calculations based on DFT to investigate the interaction of inclusion complexes between 4-NP and α-, β-, and γ-CDs. The results revealed that α-CD forms the most stable and energetically favorable inclusion complex with 4-NP, followed by β-CD, while γ-CD exhibited lower affinity due to steric limitations. The three approaches converged on the predominance of physical adsorption as the primary interaction mechanism, with hydrogen bonding and van der Waals forces playing central roles in stabilizing complex systems. The integration of these theoretical methodologies provides valuable theoretical insight into the selective encapsulation behavior of cyclodextrins. It highlights α-CD as an up-and-coming candidate for the removal of 4-NP from aqueous environments. The study emphasizes the potential of cyclodextrin-based materials as sustainable, biodegradable, and efficient adsorbents for environmental remediation and underscores their applications in wastewater treatment technologies targeting phenolic contaminants.

## References

[1] Cardoso
Juarez A. O., Ivan Ocampo Lopez E., Kesarla M. K., Bogireddy N. K. R. (2024). Advances
in 4-Nitrophenol Detection and Reduction Methods and Mechanisms: An
Updated Review. ACS Omega.

[2] Yahya A. A., Rashid K. T., Ghadhban M. Y., Mousa N. E., Majdi H. S., Salih I. K., Alsalhy Q. F. (2021). Removal
of 4-Nitrophenol from Aqueous
Solution by Using Polyphenylsulfone-Based Blend Membranes: Characterization
and Performance. Membranes.

[3] Feng W., Deng Y., Yang F., Miao Q., Ngien S. K. (2023). Systematic
Review of Contaminants of Emerging Concern (CECs): Distribution, Risks,
and Implications for Water Quality and Health. Water.

[4] Musuc A. M. (2024). Cyclodextrins:
Advances in Chemistry, Toxicology, and Multifaceted Applications. Molecules.

[5] Alsbaiee A., Smith B. J., Xiao L., Ling Y., Helbling D. E., Dichtel W. R. (2016). Rapid Removal of
Organic Micropollutants from Water
by a Porous β-Cyclodextrin Polymer. Nature.

[6] Syeda S. E. Z., Nowacka D., Khan M. S., Skwierawska A. M. (2022). Recent
Advancements in Cyclodextrin-Based Adsorbents for the Removal of Hazardous
Pollutants from Waters. Polymers.

[7] Wacławek S., Krawczyk K., Silvestri D., Padil V. V. T., Řezanka M., Černík M., Jaroniec M. (2022). Cyclodextrin-based
Strategies for Removal of Persistent Organic Pollutants. Adv. Colloid Interface Sci..

[8] Urooj T., Mishra M., Pandey S. (2024). Unlocking
Environmental Solutions:
A Review of Cyclodextrins in Pollutant Removal. Discover Environment.

[9] Saini S. S., Mebert A., Copello G. J. (2025). A Novel,
Green, β-Cyclodextrin-Based
Microextraction and Enrichment Method for Sensitive HPLC-UV Determination
of 4-Nitrophenol in Groundwater. ChemistrySelect.

[10] Al-Absi R. S., Al-Qodah Z., Al-Shannag M., Al-Busoul M. F., Al-Kharabsheh M. A., Al-Harahsheh M. S. (2025). Cyclodextrin-Based
Nanosponges for
the Removal of Heavy Metals from Aqueous Solutions: A Review. Applied Sciences.

[11] Erdős M., Hartkamp R., Vlugt T. J. H., Moultos O. A. (2020). Inclusion Complexation
of Organic Micropollutants with β-Cyclodextrin. J. Phys. Chem. B.

[12] Ma L., Chen Y., Wang Z. (2024). Computational Insights
into Cyclodextrin Inclusion Complexes with the Organophosphorus Flame
Retardant DOPO (α-, β-, γ-CD; docking + MD + QM). Molecules.

[13] Benmerabet A., Bouhadiba A., Belhocine Y., Rahali S., Sbei N., Seydou M., Boucheriha I., Omeiri I., Assaba I. M. (2023). DFT Investigation
on the Complexation of β-Cyclodextrin and Hydroxypropyl-β-Cyclodextrin
as Recognition Hosts with Trichloroethylene. Atoms.

[14] Kiselev S. S., Borisov Y. A. (2016). Complexes of α-,
β-, and γ-cyclodextrins
with nitrophenols: A theoretical study of the structure and energy. Journal of Structural Chemistry.

[15] Khuttan S., Azimi S., Wu J. Z., Dick S., Wu C., Xu H., Gallicchio E. (2023). Taming multiple
binding poses in alchemical binding
free energy prediction: the β-cyclodextrin host–guest
SAMPL9 blinded challenge. Phys. Chem. Chem.
Phys..

[16] Procacci P., Guarnieri G. (2022). Binding Free
Energies of β-Cyclodextrin Complexes
from Nonequilibrium Alchemical Simulations: The Role of Host Flexibility. J. Chem. Theory Comput..

[17] Zhang X., Li H., Xu J., Wang L. (2023). Exploring
Host–Guest Interactions
in Cyclodextrin Complexes through NCI and AIM Analyses: Insights into
Noncovalent Stabilization Mechanisms. J. Mol.
Struct..

[18] Hohenberg P., Kohn W. (1964). Inhomogeneous Electron
Gas. Phys. Rev..

[19] Kohn W., Sham L. J. (1965). Self-Consistent
Equations Including Exchange and Correlation
Effects. Phys. Rev..

[20] Soler J. M., Artacho E., Gale J. D., García A., Junquera J., Ordejón P., Sánchez-Portal D. (2002). The SIESTA
method for ab initio order-N materials simulation. J. Phys.: Condens. Matter.

[21] Perdew J. P., Zunger A. (1981). Self-interaction correction
to density-functional approximations
for many-electron systems. Phys. Rev. B.

[22] Boys S. F., Bernardi F. (1970). The calculation of
small molecular interactions by
the differences of separate total energies. Some procedures have reduced
errors. Mol. Phys..

[23] Rubim A. M., Rubenick J. B., Vendrame L. O., Zanella I., Rolim C. M. B., Rhoden C. R. B. (2024). Formulation and
Characterization of Amiodarone–Methyl-β-Cyclodextrin
Inclusion Complexes: A Molecular Modelling Perspective. J. Mol. Graph. Model..

[24] Tonel M., Schemmer J., Vendrame L., Vendrame L. F. O., Zanella I., Fagan S. (2024). Phthalates Adsorption on Nanostructures
for Environmental Remediation:
An ab Initio Study. J. Braz. Chem. Soc..

[25] Trott O., Olson A. J. (2010). AutoDock Vina: Improving the Speed
and Accuracy of
Docking with a New Scoring Function, Efficient Optimization, and Multithreading. J. Comput. Chem..

[26] Eberhardt J., Santos-Martins D., Tillack A. F., Forli S. (2021). AutoDock Vina 1.2.0:
New Docking Methods, Expanded Force Field, and Python Bindings. J. Chem. Theory Comput..

[27] González-Durruthy M., Concu R., Vendrame L. F. O., Ortiz Martins M., Zanella I., Ruso J. M., Cordeiro M. N. D. S. (2022). Computational
Modeling on Binding Interactions of Cyclodextrins with the Human Multidrug
Resistance P-Glycoprotein Toward Efficient Drug-Delivery System Applications. Curr. Top. Med. Chem..

[28] Schrödinger, LLC . The PyMOL Molecular Graphics System, Version 2.5. Schrödinger, LLC: New York, NY, 2021.

[29] Discovery Studio: Dassault Systèmes BIOVIA. BIOVIA Discovery Studio Modeling Environment, Release 2021. Dassault Systèmes BIOVIA: San Diego, CA, USA, 2021.

[30] Kirschner K. N., Yongye A. B., Tschampel S. M., González-Outeiriño J., Daniels C. R., Foley B. L., Woods R. J. (2008). GLYCAM06: A Generalizable
Biomolecular Force Field. J. Comput. Chem..

[31] Wang J., Wolf R. M., Caldwell J. W., Kollman P. A., Case D. A. (2004). Development
and Testing of a General Amber Force Field. J. Comput. Chem..

[32] Wang J., Wang W., Kollman P. A., Case D. A. (2006). Automatic Atom Type
and Bond Type Perception in Molecular Mechanical Calculations. J. Mol. Graph. Model..

[33] Bayly C. I., Cieplak P., Cornell W., Kollman P. A. (1993). A wellbehaved
electrostatic
potential-based method using charge restraints for deriving atomic
charges: The RESP model. J. Phys. Chem..

[34] Frisch, M. J. ; Trucks, G. W. ; Schlegel, H. B. ; Scuseria, G. E. ; Robb, M. A. ; Cheeseman, J. R. ; Scalmani, G. ; Barone, V. ; Petersson, G. A. ; Nakatsuji, H. ; Gaussian 16, Revision C.01.

[35] da
Rocha J. A. P., da Costa R. A., da Costa A. d. S. S., da Rocha E. C. M., Gomes A. J. B., Machado A. K., Fagan S. B., Brasil D. d. S. B., Lima e Lima A. H. (2024). Harnessing Brazilian Biodiversity
Database: Identification of Flavonoids as Potential Inhibitors of
SARS-CoV-2 Main Protease Using Computational Approaches and All-Atom
Molecular Dynamics Simulation. Front. Chem..

[36] Rocha J. A. P. d., da Costa R. A., Rocha E. C. M. d., Machado A. K., Bick D. L. U., Fagan S. B., Wanzeller A. L. M., Gomes M. D. d. S., Rodrigues J. L. L., Rego J. d. A. R. d., Brasil D. d. S. B., Lima A. H. (2025). Antiviral Effect of Piperine on Chikungunya Virus:
In Vitro Evidence and In Silico Analysis of E1-E2 Binding. ACS Omega.

[37] da
Costa R. A., da Rocha J. A. P., Pinheiro A. S., da Costa A. S. S., da Rocha E. C. M., Josino L. P. C., da
Gonçalves A. S., Lima A. H. L., Brasil D. S. B. (2022). In silico identification
of novel allosteric inhibitors of Dengue virus NS2B/NS3 serine protease. J. Serb. Chem. Soc..

[38] Saini S. S., Copello G. J., Fagan S. B., Tonel M. Z. (2023). Comparison of three
cyclodextrins to optimize bisphenol A extraction from source water:
Computational, spectroscopic, and analytical studies. J. Sep. Sci..

[39] Eftink M. R., Andy M. L., Bystrom K., Perlmutter H. D., Kristol D. S. (1989). Cyclodextrin inclusion complexes: studies of the variation
in the size of alicyclic guests. J. Am. Chem.
Soc..

[40] Cromwell W. C., Bystrom K., Eftink M. R. (1985). Cyclodextrin-adamantanecarboxylate
inclusion complexes: studies of the variation in cavity size. J. Phys. Chem..

[41] Tang P., Sun Q., Suo Z., Wang X., Wang C., Zhang B. (2018). Rapid and
efficient removal of estrogenic pollutants from water by using beta-
and gamma-cyclodextrin polymers. Chem. Eng.
J..

[42] Szejtli J. (1998). Introduction
and General Overview of Cyclodextrin Chemistry. Chem. Rev..

[43] Mandeep, Sharma L., Kakkar R. (2018). DFT study
on the adsorption of p-nitrophenol over vacancy and Pt-doped graphene
sheets. Comput. Theor. Chem..

[44] Gandhimathi R., Dheivamalar S., Dhanasekaran R. (2015). Geometry optimization, HOMO and LUMO
energy, molecular electrostatic potential, NMR, FT-IR and FT-Raman
analyzes on 4-nitrophenol. Eur. Phys. J. Appl.
Phys..

[45] Cramer F., Saenger W., Spatz H. C. (1967). Inclusion
Compounds. XIX. 1a The
Formation of Inclusion Compounds of α-Cyclodextrin in Aqueous
Solutions. Thermodynamics and Kinetics. J. Am.
Chem. Soc..

[46] Pendergast D. D., Connors K. A. (1985). Complexes of disubstituted benzene positional isomers
with α-cyclodextrin. Bioorg. Chem..

[47] Connors K. A., Pendergast D. D. (1984). Microscopic
binding constants in cyclodextrin systems:
complexation of α-cyclodextrin with sym-1,4-disubstituted benzenes. J. Am. Chem. Soc..

[48] Celebioglu A., Topuz F., Yildiz Z. I., Uyar T. (2019). Efficient Removal of
Polycyclic Aromatic Hydrocarbons and Heavy Metals from Water by Electrospun
Nanofibrous Polycyclodextrin Membranes. ACS
Omega.

[49] Yu Z., Grasso M. F., Sorensen H. H., Zhang P. (2019). Ratiometric SERS detection
of polycyclic aromatic hydrocarbons assisted by β-cyclodextrin-modified
gold nanoparticles. Microchim. Acta.

[50] Rekharsky M. V., Inoue Y. (1998). Complexation Thermodynamics
of Cyclodextrins. Chem. Rev..

[51] de
Namor A. F. D., Blackett P. M., Cabaleiro M. C., Al Rawi J. M. A. (1994). Cyclodextrin–monosaccharide interactions in
water. J. Chem. Soc., Faraday Trans..

[52] Godínez L. A., Schwartz L., Criss C. M., Kaifer A. E. (1997). Thermodynamic Studies
on the Cyclodextrin Complexation of Aromatic and Aliphatic Guests
in Water and Water–Urea Mixtures. Experimental Evidence for
the Interaction of Urea with Arene Surfaces. J. Phys. Chem. B.

[53] Schneiderman E., Stalcup A. M. (2000). Cyclodextrins: a versatile tool in separation science. J. Chromatogr. B: Biomed. Sci. Appl..

[54] Zolfaghari G. (2016). β-Cyclodextrin
incorporated nanoporous carbon: Host–guest inclusion for removal
of p-Nitrophenol and pesticides from aqueous solutions. Chem. Eng. J..

[55] László K., Podkościelny P., Da̧browski A. (2003). Heterogeneity
of Polymer-Based Active
Carbons in Adsorption of Aqueous Solutions of Phenol and 2,3,4-Trichlorophenol. Langmuir.

[56] Jitapunkul K., Toochinda P., Lawtrakul L. (2021). Molecular Dynamic Simulation Analysis
on the Inclusion Complexation of Plumbagin with β-Cyclodextrin
Derivatives in Aqueous Solution. Molecules.

[57] Alvira E. (2018). Theoretical
Study of the β-Cyclodextrin Inclusion Complex Formation of Eugenol
in Water. Molecules.

[58] Sancho M. I., Andujar S., Porasso R. D., Enriz R. D. (2016). Theoretical and
Experimental Study of Inclusion Complexes of Chalcone and 2′,4′-Dihydroxychalcone
with β-Cyclodextrin. J. Phys. Chem. B.

